# Less Negative Implicit Attitudes Toward Autism Spectrum Disorder in University Students: A Comparison with Physical Disabilities

**DOI:** 10.1007/s10803-022-05749-y

**Published:** 2022-10-15

**Authors:** Susumu Yokota, Mari Tanaka

**Affiliations:** Faculty of Arts and Science, Kyushu University, 744 Motooka, 819-0395 Nishiku, Fukuoka Japan

**Keywords:** Implicit attitude, Explicit attitude, Autism spectrum disorder, Public stigma, Implicit association test, Social desirability

## Abstract

People with autism spectrum disorder (ASD) experience stigmatization rooted in negative attitudes or prejudice toward them due to social awkwardness. However, little is known about implicit attitudes toward ASD, especially differences in attitudes compared to those of more visible conditions; physical disabilities. In this study, we implemented implicit association tests (IATs) to assess implicit attitudes. Sixty-three university students participated in IATs and answered questionnaires that measured explicit attitudes, social desirability, knowledge about—and familiarity with—disorders. The results demonstrated that implicit attitude toward ASD was significantly less negative than toward physical disabilities. Regarding the discrepancy, not socially awkward behavior but appearance of people with ASD can be evaluated as ‘in-group’ members and lead to less negative attitude compared with physical disabilities.

Although the prevalence of autism spectrum disorder (ASD) has varied among studies, approximately 18.5 per 1000 children are diagnosed with ASD (Maenner et al., 2020). In recent times, the number of university students with ASD has increased (Bakker et al., 2019). Although enrollment is the first step in inclusive education in universities, students with ASD are stigmatized and face several difficulties, such as exclusion or discrimination in personal relationships throughout their school experience (Cappadocia et al., 2012; Hwang et al., 2018; Wiorkowski, 2015). Both individual factors (such as communication difficulties or anxiety) and contextual factors (fewer friends at school) comprise risk factors related with such stigmatization (Cappadocia et al., 2012). In this study, we focused on attitudes toward people with ASD as a contextual risk factor.

Attitude (also termed “prejudice”) is defined as the evaluation of—or affect associated with—a certain social group (Greenwald, 1990; Hewstone et al., 2002). Human behavior is highly modulated by a person’s attitude toward certain things (Fazio et al., 1995; Fazio & Roskos-Ewoldsen, 2005; Stanley et al., 2008). Unlike other disabilities, such as physical disabilities for instance, some ASD traits are invisible to the general population (Zeedyk et al., 2019). As the appearance of people with ASD does not differ from neurotypical people, other people tend to expect the former to behave like the latter. However, autistic individuals may be judged by their social behaviors rather than physical appearance. For instance, Aubé et al., (2021) argued that this gap between social expectations and the actual behavior of people with ASD precipitates negative attitudes and exclusion. Butler & Gillis (2011) reported that atypical social behavior significantly negatively impacted attitude, while the label of ASD did not. Contrary to this finding, Iobst et al., (2009), provided labeling or background information to participants, revealed that labeling had a negative impact, but explanatory or neuropsychological background information improved their attitude.

Previous studies have revealed the factors that affect these attitudes—namely, participants’ sex, knowledge about ASD, quality or quantity of contact with people with ASD, academic program, and individual traits (Gardiner & Iarocci, 2014; Matthews et al., 2015; White et al., 2019). Mahoney (2007) investigated these factors based on three aspects of attitude: behavioral, cognitive, and emotional. This study revealed relatively positive attitudes toward ASD among university students, and participants’ sex, knowledge, and quality of contact were significantly associated with attitudes toward ASD. Regarding knowledge and contact experience, Nevill & White (2011) revealed that students who reported having a relative with ASD were significantly more open to interacting with students with ASD. Similarly, White et al. (2019) conducted a five-year cohort study and revealed a positive change in attitudes toward ASD and improvement in knowledge about ASD. Moreover, intervention studies using online training have reported that increasing knowledge could decrease negative attitudes toward people with ASD (Gillespie-Lynch et al., 2015). Online training included current research about ASD (e.g., changes in diagnostic criteria, early signs of ASD, or etiology).

Previous studies have examined attitudes using self-evaluating questionnaires—that is, explicit measurement. Explicit measures have been frequently associated with the social desirability bias, that is, respondents’ tendency to provide more socially accepted answers (Crowne & Marlowe, 1960; Krumpal, 2013). Respondents might infer what researchers intend to investigate from explicit measures. Therefore, they may pretend to demonstrate positive attitudes toward the target group irrespective of their real attitude. Indeed, attitudes toward persons with disabilities evaluated by explicit measurements are distorted to align with more socially desirable directions (Hinshaw & Stier, 2008; Pruett & Chan, 2006). Previous findings regarding explicit attitudes toward ASD have been largely underestimated.

In this study, we focused on implicit attitudes (also termed “prejudice”), which manifest as actions or judgments that are under the control of automatically activated evaluation, without the performer’s awareness of the causation (Greenwald & Banaji, 1995). While explicit attitudes are related to more controllable behavior, such as friendliness, implicit attitudes toward certain group members are significantly associated with more subtle discriminative behaviors, such as interpersonal distancing, behavioral rejection, or causing discomfort (Dovidio et al., 2002; Lipson et al., 2020).

Recently, a growing number of studies have examined implicit attitudes of children (Aubé et al., 2021), university students (Jones et al., 2021; Lipson et al., 2020; Obeid et al., 2021), and parents of children with ASD (Dickter et al., 2021) toward ASD. Lipson et al., (2020) examined university students’ perceptions of—and behavior toward—student confederates with or without ASD using an implicit association test (IAT; Greenwald & Banaji 1995). They found a significant negative attitude toward ASD and an association between implicit attitudes and some behaviors toward student confederates with ASD compared to those without ASD. Obeid et al., (2021) also implemented IATs and revealed that negative implicit attitudes toward ASD in university students was correlated with insufficient knowledge about—and lesser pleasant experiences with—people with ASD.

Despite the growing number of studies examining implicit attitudes toward ASD, a large part of the previous studies have conducted comparisons with typically developing people without other disorders or diseases. For example, Thibodeau & Finley (2017) compared implicit attitudes toward the mother of a child with ASD and the mother of a child with asthma. They found no group differences in explicit attitudes but a significantly less positive implicit attitudes toward the mother of a child with ASD compared to mothers with a child with asthma. However, considering the invisibility of some ASD traits from the general population, it is important to compare implicit attitudes toward persons with more visible conditions, such as physical disabilities, including the physically handicapped or blind. Although previous studies on attitudes toward physical disabilities have revealed moderate to strong negative implicit attitudes (Aaberg, 2012; Chen et al., 2011; Wilson & Scior, 2014), whether attitudes toward a certain disorder generalize to other types of disorder, especially in terms of the invisibility of some traits of the disorder, is still unclear.

This study aimed to delineate implicit and explicit attitudes toward ASD by comparing them with those toward physical disabilities and its related factors, such as knowledge and contact with persons with disabilities. To this end, we implemented two behavioral IATs to measure implicit attitudes and questionnaires to evaluate explicit attitudes. We also examined the effect of the social desirability bias on these two measurements. We hypothesized that social desirability bias was not related with implicit attitudes, but related with explicit attitudes. We also expected that university students generally exhibit positive explicit attitudes and negative implicit attitudes. Regarding the generalization of the attitudes toward disorders, we hypothesized that participants would show different implicit attitudes toward ASD than toward physical disabilities because of the invisibility of some ASD traits and the contrast between appearance and behaviors.

## Methods

### Participants

Sixty-three Japanese university students participated in this study. The sample included 31 male students and 32 female students. Participants’ age ranged from 18.2 to 25.5, with a mean age of 20.2 years. All participants indicated they did not have any disabilities. Participants were recruited through university classes and mailing lists. Participants who were recruited through the classes were informed that participation was completely independent from course credits. Additionally, some of them also participated in another project that examined the neural basis of implicit attitudes.

This study was approved by the Ethics Committee of the Kyushu University Faculty of Arts and Science. Prior to the experiment, written informed consent was obtained from all participants or their parents if they were under 20 years of age, in accordance with the Declaration of Helsinki.

### Measures and procedures

#### Implicit Attitudes

We implemented a computer-based IAT to assess implicit attitudes toward ASD (A-IAT) and physical disabilities (P-IAT). In this computer-based task, participants have to categorize the target picture/word using one of two keys along two dimensions. One dimension involves target concepts (e.g., “disorder” and “healthy”), and the other relates to attribute concepts (e.g., “good” and “bad”). Categories consist of a pair of these concepts and share a response key. For example, if “disorder” and “good” are combined and placed to the left corner of the screen, participants have to categorize pictures associated with “disorder” and “good” words into the left category by pressing the corresponding key. Responses are faster and more accurate when strongly associated categories that share the same response key (e.g. “disorder” and “bad”) (Lane et al., 2007).

The IATs were administered according to standard procedures (Greenwald et al., 1998), with the stimulus composed of pictures and words. Participants classified pictures relating to ASD vs. healthy for the A-IAT and physical disabilities vs. healthy for the P-IAT. Words were always classified as good or bad in import. To reduce effects of race, ethnicity, or valence to the attitudes toward disorders, we used pictograms depicting the features of the disorders selected from the website. All words were Japanese adjectives chosen from the Balanced Corpus of Contemporary Written Japanese (BCCWJ; https://clrd.ninjal.ac.jp/bccwj/en/index.html).

We conducted a pilot study on stimulus selection, which comprised 20 university students. The participants for this pilot study were not included in the main study. In the pilot study, we prepared pictograms of 51 individuals with ASD, 30 with physical disabilities, and 30 healthy persons. These pictograms were selected from websites for the enlightenment campaign of ASD or free illustration sites. In Japan, the administrative classification of physical disabilities includes not only physical impairments, such as difficulty walking or using the wheelchair, but also visual and hearing impairments. Therefore, we selected pictures depicting physical and visual impairments, which were more evident from their appearance among these physical disabilities.

**Fig. 1:**
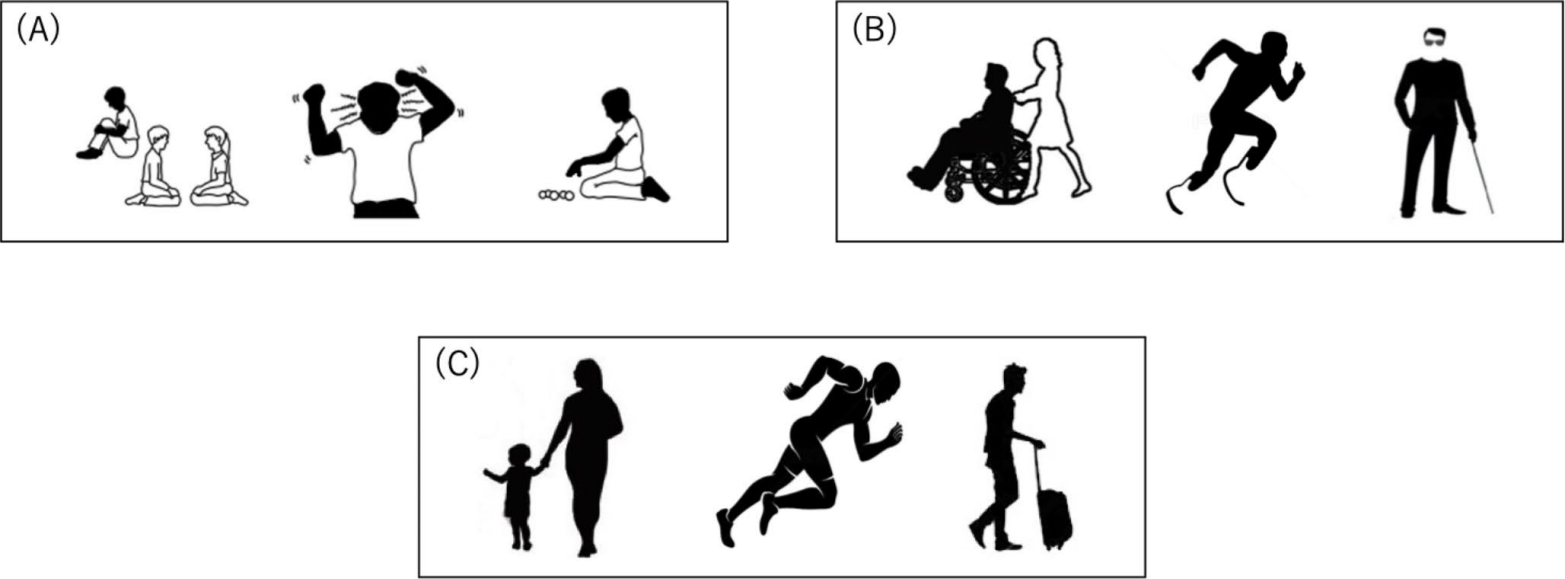
*Examples of the picture set* In the implicit association test for ASD, (a) and (c) were used as stimulus, and depicted ASD and healthy person, respectively. In test for physical disabilities, (b) and (c) were used as stimulus, and depicted physical disabilities and healthy person, respectively

For picture selection, participants judged whether the presented pictogram depicted a person with ASD or a healthy individual for the A-IAT, and a person with physical disability or a healthy individual for the P-IAT. Based on the frequency in the BCCWJ, the words chosen were 200 words that were most frequently used in daily life. Participants judged each presented word as good or bad. From the results of the pilot study, we chose the 20 most frequently categorized pictures to represent the ASD, physical disabilities, and healthy groups (Fig. 1), and 20 words (e.g., “happy,” “beautiful,” “delight,” “ugly,” or “dirty”) implying good and bad meanings.

**Fig. 2:**
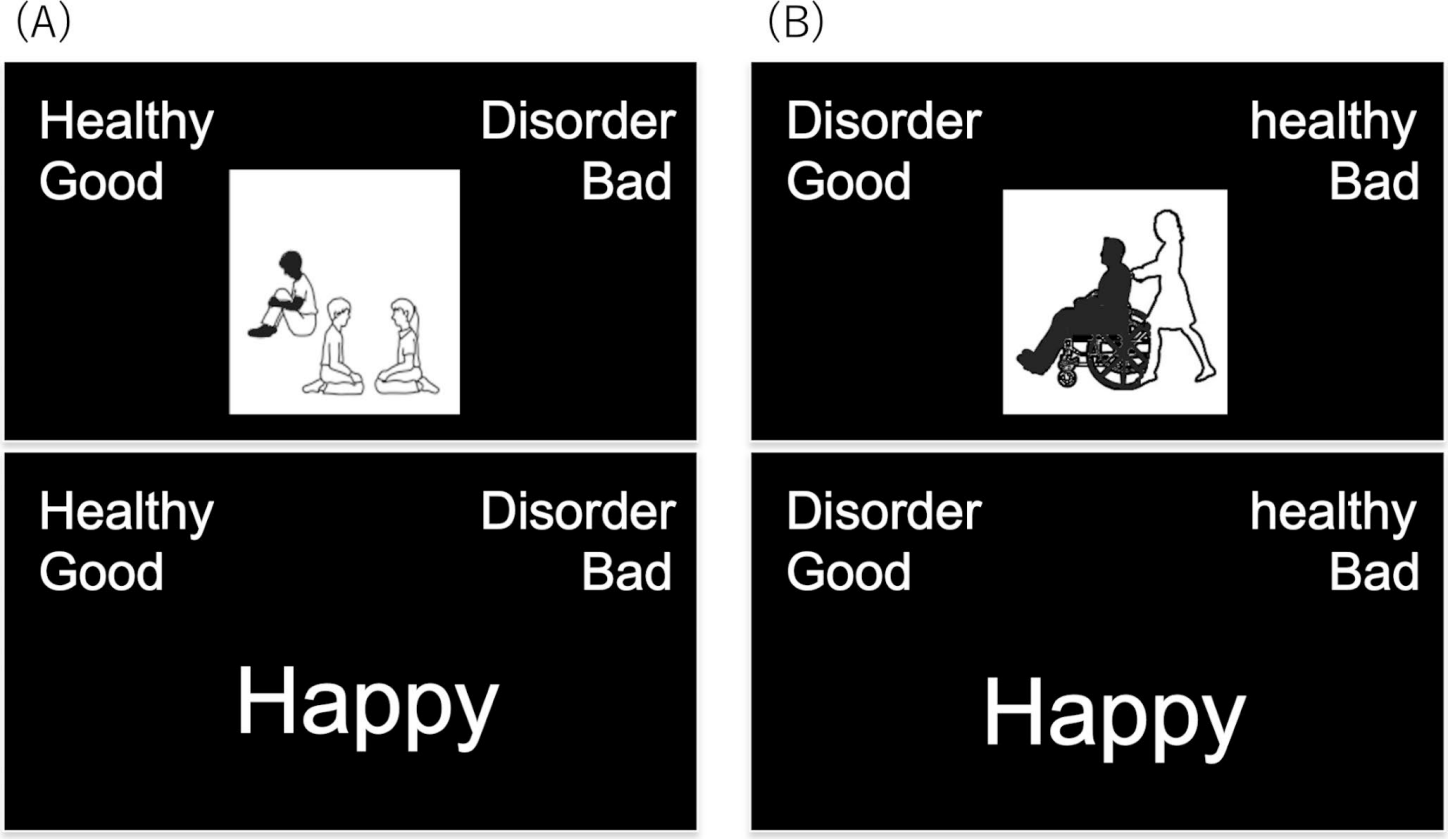
*Visual representation of implicit association test* In the congruent condition, “disorder and bad” and “healthy and good” were placed in both upper corners of the screen in pairs. In the incongruent condition, these were placed in pairs. The order of conditions and positions of categories were counterbalanced among the participants. (A) Congruent condition of the A-IAT and (B) incongruent condition of the P-IAT

Both IATs had seven blocks and two conditions: congruent and incongruent conditions (Table 1). Blocks 1, 2, and 5 were the training blocks. Each training block consisted of 20 trials. In blocks 1 and 5, participants categorized pictures along the dimensions of “disorder” or “healthy.” The names of the categories for pictures were the same in both tasks (“disorder” and “healthy”). Therefore, participants were requested to categorize pictures depicting ASD in the A-IAT and physical disability in the P-IAT as “disorder,” and pictures depicting persons without disabilities as “healthy.” In Block 2, words had to be categorized as good or bad. There were 40 trials in the test blocks (blocks 3, 4, 6, and 7). In these blocks, pictures or words were presented randomly, and participants categorized pictures into “disorder or healthy,” and words into “good or bad.” Categories were always shown in both the upper corners of the screen. In the congruent condition, the “disorder and bad” and the “healthy and good” categories were always shown in pairs. In the incongruent condition, the “disorder and good” and “healthy and bad” categories were paired. The order of conditions and positions of categories were counterbalanced across participants (Fig. 2).

**Table 1:** *Sequence of Trial Blocks in IATs*

Congruent condition ^a^				
Block	N of trials	Stimulus	Left category ^a^	Right category ^a^
1	20	Pictures	Healthy	Disorder
2	20	Words	Good	Bad
3	20	Pictures + Words	Healthy + Good	Disorder + Bad
4	40	Pictures + Words	Healthy + Good	Disorder + Bad
Incongruent condition ^a^				
Block	N of trials	Stimulus	Left category ^a^	Right category ^a^
5	20	Pictures	Disorder	Healthy
6	20	Pictures + Words	Disorder + Good	Healthy + Bad
7	40	Pictures + Words	Disorder + Good	Healthy + Bad

^a^: Order of condition and category position were counterbalanced among participants

We predicted that some participants would be unfamiliar with—and possessed poor knowledge about—people with ASD. Gillespie-Lynch et al., (2015) reported that misconceptions of ASD were common among university students. They also commonly confused ASD with other disorders, such as learning disabilities. Therefore, to reduce responses generated from misconceptions and confusions, participants were requested to read 13 passages on ASD before running the tasks (e.g., “People with ASD tend to indicate abnormal social approach or one-way manner conversation”; “People with ASD have trouble making friends”). These passages were created consistently with the behavioral criteria or traits of the Diagnostic and Statistical Manual of Mental Disorders fifth edition (DSM-5; American Psychiatric Association 2013).

The tasks were presented using presentation software (Neurobehavioral Systems, USA) running on a mobile PC (VAIO; Sony, Japan). Response time and answers were recorded using the software. The participants responded by pressing a button. When the targeted picture or word belonged to a category on the right-hand-side, they were instructed to press the J key, and when the target belonged to the left-hand-side category, they were instructed to press the F key. If they answered incorrectly, a prompt appeared for them to correct their response.

### Questionnaires

#### Explicit Attitudes

We used the Attitude Toward Disabled Persons scale (ATDP) Form O to measure explicit attitudes. The ATDP was developed as a unidimensional measure of attitudes toward people with disabilities (Yuker et al., 1970). In the present study, we did not modify the original wording “people with disabilities,” but instructed participants to answer about people with physical disabilities for measuring attitudes toward physical disabilities and people with ASD for measuring attitudes toward people with ASD, respectively. The scale contains 20 items rated on a 6-point Likert scale ranging from + 3 (= I agree very much) to -3 (= I disagree very much). Test-retest reliability of this scale ranged from 0.71 to 0.83.

#### Social Desirability

Tani (2008) translated Paulhus’s (1991) Balanced Inventory of Desirable Responding into Japanese and confirmed the psychometric validity of the Japanese version of the Balanced Inventory of Desirable Responding (BIDR-J). This scale comprises 24 items rated on a 5-point Likert scale ranging from 1 (= disagree) to 5 (= agree). The scale has two factors: self-deception, where the respondent actually believes their positive self-reports, and impression management, where the respondent consciously dissembles (Paulhus, 1984). The two factors had 12 items each and internal consistency values of 0.75 and 0.70. Consistency value of the total scale was 0.72 (Tani, 2008).

#### Knowledge of and Experience with Persons with Disabilities

We evaluated participants’ perceptions of their knowledge of ASD, visual impairments, and hearing impairments. Participants answered one question for each condition related to the extent of their knowledge from 1 (= do not know at all) to 5 (= know very well). As for their related experience, participants answered simple yes/no questions that inquired whether they had contacted a person with the particular disability.

### Data Treatment and Scoring

#### Implicit Attitude

We calculated D scores from the response time of each test block (blocks 3, 4, 6, and 7) in both the A-IAT and P-IAT separately. We adopted the D_6_ algorithm for calculations (Greenwald et al., 2003). In the upper and lower tail treatments, trials with response times greater than 10,000 ms and less than 400 ms were eliminated. The response time of error trials was replaced with the block mean of the correct response time + 600 ms penalty time. Then, the mean reaction time of the incongruent condition was subtracted from that of the congruent condition. This difference was divided by the standard deviation (SD) of the reaction time under both conditions. Therefore, a positive value of the D score reflected the strong association between the categories “disorder” and “bad,” indicating a negative implicit attitude.

#### Explicit Attitude

Using Yuker et al.’s (1970) scoring method, we calculated the ATDP score. After changing the signs of some reverse items (items 2, 5, 6, 11, and 12), we summed each response and changed the sign of the sum. Finally, we added a constant of 60; thus, the total score ranged from 0 to 120. The neutral value was considered to be 60.

#### Social Desirability

As proposed by Tani (2008), we calculated the total score and factor scores (self-deception and impression management) in the BIDR-J. Due to some misarrangements in the procedure, we did not obtain the BIDR-J scores from 10 participants. Thus, we included 53 participants in further analyses related to the BIDR-J.

### Data Analysis

#### Implicit Attitude

We conducted a one-sample t-test using D scores of both the A-IAT and P-IAT to confirm whether implicit attitudes toward ASD and physical disabilities were positive. As described above, the D scores were calculated from the incongruent minus congruent condition. We checked whether the D scores were significantly different from 0. Along with some previous studies (Hein et al., 2011; Takahashi et al., 2009), we also conducted a two-way analysis of covariance (ANCOVA) using reaction time in each condition (congruent or incongruent) and disability (ASD or physical disability) to compare the differences between conditions directly. Age, sex, and the score of impression management in the BIDR-J were treated as covariates.

#### Explicit Attitude

We conducted a one-sample t-test using the ATDP to confirm whether explicit attitudes toward ASD and physical disabilities were positive. We tested whether each score was statistically different from 60. We also conducted a dependent t-test to compare the explicit attitudes toward ASD and physical disabilities.

#### Relationships among Attitude, Social desirability, Knowledge, or Experience

Each score used in the correlation analysis was standardized for mean 0, SD 1. We then calculated Pearson’s correlation coefficients between attitudes and the BIDR-J. Similarly, we also conducted Pearson’s correlation between attitudes and knowledge toward the disabilities. As for the experience with a person with disability, we divided participants according to whether or not they had contact with a person with a disability. We then conducted an independent t-test to compare these group differences in implicit and explicit attitudes. All statistical analyses were conducted using the SPSS ver. 24 (IBM, Japan) and R.

## Results

### Implicit Attitude

**Fig. 3:**
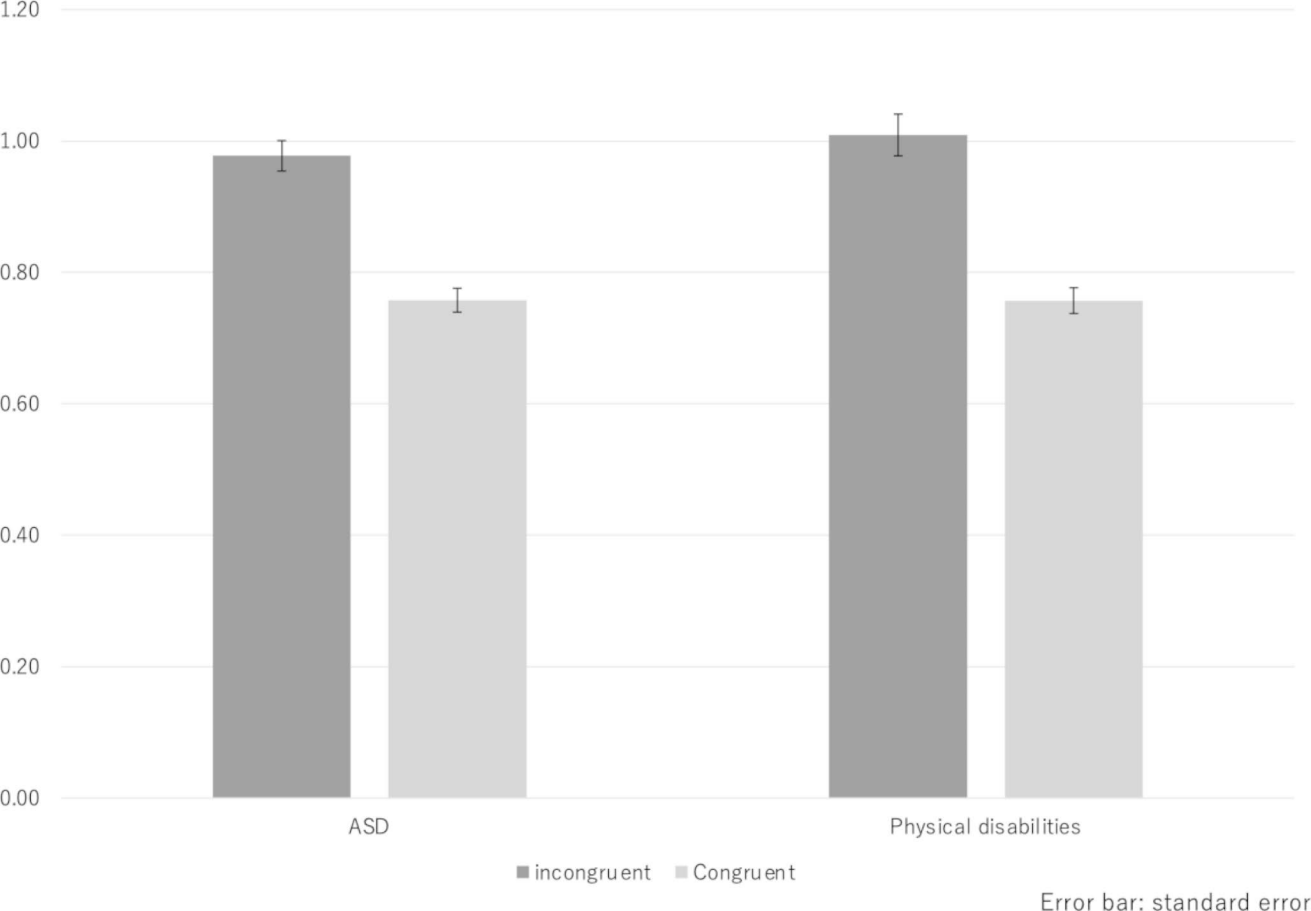
*Mean reaction time of implicit association tests* A two-way ANCOVA revealed significant main effects of disorder and consistency. Reaction times (RT) for physical disabilities were significantly longer than those for ASD. We also found significant main effects of consistency. The RTs of the incongruent condition were significantly longer than those of the congruent conditions

The summary of the variables is shown in Table 2. One-sample t-tests revealed that both D scores were significantly different from 0 (ASD: *t*(62) = 11.6, *p* < 0.001; physical disabilities: *t*(62) = 14.8, *p* < 0.001). Since both D scores comprised positive values, it meant that participants exhibited negative implicit attitudes toward both ASD and physical disabilities. To investigate the response tendency more precisely, we conducted a two-way ANCOVA. The results showed a significant main effect of disability (*F*(1, 49) = 4.2, *p* < 0.05, *ω*^*2*^ = 0.02) and consistency (*F*(1, 49) = 23.9, *p* < 0.001, *ω*^*2*^ = 0.10). We did not find a significant interaction between disability and consistency (*F*(1, 49) = 0.8, *p* = 0.38). These results revealed that the reaction time of the P-IAT was longer than that of the A-IAT, and the reaction time of the incongruent condition was significantly longer than that of the congruent condition in both IAT tasks (Fig. 3).

**Table 2:** *Descriptive Statistics of the IATs and Questionnaires*

Variables			Mean	SD
A-IAT				
	D Score		0.58^a^	0.39
	RT	Incongruent	0.98	0.17
		Congruent	0.76	0.13
P-IAT				
	D Score		0.62 ^a^	0.33
	RT	Incongruent	1.01	0.23
		Congruent	0.76	0.14
ATDP				
	ASD		65.49 ^a^	11.18
	Physical disabilities		67.11 ^a^	11.58
Social desirability				
		Self-deception	33.81	5.35
		Impression management	38.19	6.171
		Total score	72	9.556
Knowledge				
		ASD	3.38	0.869
		Visual impairments	3.27	0.865
		Hearing impairments	3.03	0.861

Abbreviations: IAT=implicit association test; ASD=autism spectrum disorder; ATDP=Attitude Toward Disabled Persons scale

a: one sample t tests revealed significant difference from neutral value (D score = 0, ATDP = 60).

## Explicit Attitude

A one-sample t-test revealed that both ATDP scores were significantly different from the neutral score of 60 (ASD: *t*(62) = 3.9, *p* < 0.001; physical disabilities: *t*(62) = 4.9, *p* < 0.001). This result indicated that participants exhibited positive explicit attitudes toward both disabilities. As for the comparison between ASD and physical disabilities, we did not find a significant difference (*t*(62) = -0.8, *p* = 0.12).

## Relationship Between Attitudes and Social Desirability

Table 3 presents the results of the correlation analyses. Pearson’s correlation coefficients were calculated using the D scores, ATDP, and BIDR-J. On the one hand, we did not find a significant correlation between the D scores and BIDR-J scores (A-IAT: self-deception; *r* = 0.06, *p* = 0.67; impression management: *r* = -0.1, *p* = 0.48; total BIDR-J score: *r* = -0.03, *p* = 0.83, P-IAT: self-deception; *r* = -0.01, *p* = 0.93; impression management: *r* = -0.1, *p* = 0.5; total BIDR-J score; *r* = -0.07, *p* = 0.62). On the other hand, we found a significant correlation between the ATDP and BIDR-J scores in ASD (impression management: *r* = 0.37, p < 0.01; total BIDR-J score; *r* = 0.35, *p* = 0.01) and physical disabilities (impression management: *r* = 0.28, *p* < 0.05; total BIDR-J score: *r* = 0.27, *p* < 0.05).

**Table 3:** *Correlations between Implicit/Explicit Attitudes and Social Desirability*

	Self-deception	Impression management	BIDR-J Total
ASD			
D score of A-IAT	0.06	-0.1	-0.03
ATDP	0.2	0.37**	0.35*
Physical disability			
D score of P-IAT	-0.01	-0.1	-0.07
ATDP	0.16	0.28*	0.27*

Abbreviations: IAT = implicit association test; ASD = autism spectrum disorder; ATDP = Attitude Toward Disabled Persons scale

*: *p* < 0.05

**: *p* < 0.01

## Relationship Between Attitudes and Knowledge or Experience

We also conducted Pearson’s correlation analyses between knowledge of disabilities and implicit/explicit attitudes. We found no significant association between knowledge and D scores or ATDPs (Table 4).

**Table 4:** *Correlation between Implicit/Explicit Attitudes and Knowledge*

	ASD	Visual impairments	Hearing impairments
ASD			
D score	-0.13	0.08	0.11
ATDP	0.003	0.03	-0.09
Physical disability			
D score	0.02	0.16	0.23
ATDP	0.13	0.15	0.05

Abbreviations: IAT = implicit association test; ASD = autism spectrum disorder; ATDP = Attitude Toward Disabled Persons scale

To investigate the relationship between attitudes and experience with persons with disabilities, we conducted a t-test to compare these group differences in implicit and explicit attitudes. Only eight participants were included in the non-contacted group. We found no significant group differences in either the D scores (ASD: *t*(61) = 1.28, *p* = 0.2; physical disabilities: *t*(61) = 0.66, *p* = 0.51) or ATDPs (ASD: *t*(61) = 0.74, *p* = 0.46; physical disabilities: *t*(61) = 0.57, *p* = 0.57).

## Discussion

As predicted, in the present study, social desirability was not related to implicit attitudes, but was significantly related to the ATDP in both conditions. Moreover, we found significant negative implicit and positive explicit attitudes toward both ASD and physical disabilities. We also found that implicit attitude toward ASD was significantly less negative than those toward physical disabilities. We did not find significant differences between ASD and physical disabilities in explicit attitudes measured by the ATDP. Additionally, we did not find significant associations between attitudes and knowledge about or contact with persons with disabilities.

We believe we obtained negative implicit and positive explicit attitudes toward both conditions owing to the effect of social desirability bias in explicit measurements. Consistent with our hypothesis, social desirability bias was not related to implicit attitudes, but was related to explicit attitudes toward both conditions. Previous studies on implicit attitudes using the IAT found no correlation between implicit attitudes and social desirability (Egloff & Schmukle, 2002; Karpinski & Steinman, 2006; Pruett & Chan, 2006). Explicit attitudes have been found to be related to conscious concepts and knowledge through learning or experience with a person with disability; they are also easily controlled by the social desirability bias (Wilson & Scior, 2014). However, implicit attitudes measured by the IAT technique reflected mechanisms beyond our conscious control and automatic associations between the target concept and valence (Greenwald & Banaji, 1995). Therefore, we speculate that positive explicit attitudes were obtained due to the consequences of conscious control. Additionally, age-related improvements in conscious control have been demonstrated (Aubé et al., 2021; Rutland et al., 2005) examined age-related changes in implicit and explicit attitudes toward ASD in preschoolers. They found that as the participants grew, children tended to conceal their negative explicit attitudes, but they still indicated negative implicit attitudes. Considering the development of conscious control, university students tended to alter their answers more positively in explicit measurements.

Implicit attitudes toward ASD were less negative than those toward physical disabilities. We predicted that attitudes toward ASD should be evaluated as more negative due to the invisibility effect, that is, the gap between expected behavior based on the appearance of people with ASD and their actual behavior. In terms of intergroup biases, people evaluate their own group (in-group) more favorably than a non-membership group (out-group) (Hewstone et al., 2002). Categorizing oneself as a member of an in-group allows one to assimilate the traits of the in-group and increases one’s similarity to other in-group members (Turner & Reynolds, 2001). Previous studies have indicated that more discernable features, such as skin color, hair style, or foreign name, are clearly features of membership to an “out-group” (Lemi & Brown, 2019; Rudman & McLean, 2016). We interpreted the appearance—rather than the socially awkward behaviors—of people with ASD, which could be evaluated as an in-group characteristic compared to people with physical disabilities.

However, Park et al., (2003) reported that individual concerns about contagious disease predicted greater negative implicit attitudes toward physical disabilities. They explained these results using the “disease-avoidance” mechanism, according to which, individuals might maintain interpersonal distance from—or avoid a person—who poses a sort of interpersonal threat (e.g., viruses, bacteria, worms, etc.). They further found, using IAT, that individuals who are more sensitive to situations with high interpersonal threat (e.g., germs transmission) indicate stronger associations between physical disability and disease. Moreover, those who read news items about interpersonal threat exhibit a stronger association. This “disease-avoidance” model is over-inclusive and becomes active to anomalous features pertaining to persons with physical disabilities.

## Limitations

This study has some limitations. In the IAT task, participants were already aware of which pictures represented persons with ASD through instruction blocks. Given that ASD is often associated with intelligence (Jensen et al., 2016), the process of labeling ASD could itself lead to a positive evaluation. However, our study did not focus on investigating this type of “positive labeling effect,” and further research that compares labeling and invisibility effects directly might be needed.

This study primarily focused on comparing attitudes toward ASD from both implicit and explicit perspectives, and not on the relationship between attitudes and actual behavior. These discriminative behaviors include ignoring, bullying, or more subtle non-verbal behaviors, such as increased physical distancing. Therefore, future studies are needed to delineate the relationship between these kinds of discriminative behaviors in daily life and the related levels of attitudes in both implicit and explicit aspects (Sue et al., 2007).

Moreover, Jones et al., (2021) conducted a intervention study using an autism acceptance training and reported an association between adequate knowledge about ASD and lower negative attitudes toward ASD. Sasson & Morrison (2019) indicated that the first impression of the adults with ASD was more positive when accurate information about the diagnosis was provided. Therefore, as described in the introduction, increasing enrollment of students with ASD in universities can lead to increased experience with—and improved acceptance of—students with ASD among general students, staff, and faculty. Contrary to the results of previous studies, we did not find a significant correlation between implicit and explicit attitudes and knowledge of—or contact with—persons with disabilities. Regarding knowledge, we used a single item that enabled a subjective evaluation of participants’ level of knowledge about ASD and physical disabilities. However, this item reflected participants’ confidence in their knowledge. To delineate the precise relationship between implicit attitudes and knowledge, items assessing individuals’ factual knowledge of ASD, such as those in the Autism Knowledge Scale (Gillespie-Lynch et al., 2015) or Autism Knowledge Questionnaire (Kuhn & Carter, 2006), should be considered. Regarding contact with persons with disabilities, the small number of participants in the non-contacted group (n = 8) limited our investigation of group differences in the analyses.

Further, cultural differences should be noted. To the best of our knowledge, two previous studies have examined differences in the knowledge of—and stigma toward—ASD in the U.S. and Lebanon (Gillespie-Lynch et al., 2019; Obeid et al., 2015). However, no previous studies have addressed differences between East Asia and the rest of the world, and no studies have been conducted thus far to address cultural differences in implicit attitudes. Since cultural differences may impact the perception of symptoms of ASD (Caron et al., 2012; Chung et al., 2012), examining cross-cultural differences in attitudes is crucial.

Furthermore, the sample size of the current study was relatively small; this was partly due to a misarrangement in the procedure of the BIDR-J, and the participants were limited to university students. To extend the generalizability of the results, future studies should focus on a broader population in universities (such as professors, assistant professors, and faculty staff).

## Conclusion

With the number of students with ASD enrolled in universities increasing, discrimination and stigmatization toward students with ASD by other students, staff, and faculty is a concerning issue. The present results highlight implicit negative and explicit positive attitudes toward both people with ASD and those with physical disabilities. We further found a significantly less negative implicit attitudes toward ASD compared to physical disabilities. From these results, we concluded that university students tended to alter their answers more positively in explicit measurements. However, considering that explicit attitudes predict controlled behavior toward people with disabilities, implicit attitudes may predict more subtle behaviors that are difficult to consciously control (Dovidio et al., 2002). To delineate attitudes more broadly, considering both implicit and explicit facets is important. Regarding the discrepancy between implicit attitudes toward ASD and physical disabilities, recent increasing enrollment trends of students with ASD may have an impact in positively shifting implicit attitudes toward ASD.

## Declarations

### Data transparency

All data generated and/or analyzed during this study are not publicly available due to the conditions of our ethics approval. The data may be made available based on receipt of a reasonable request to the corresponding author (SY).
